# Quest for the best procedure in minimal access thoracic surgery: Optimization of what?

**DOI:** 10.4103/0972-9941.28187

**Published:** 2006-12

**Authors:** Tamas F Molnar

**Affiliations:** Department of Surgery, Medical Faculty, University of Pécs, Hungary

Originally this was to be an article about a single surgeon’s experience of minimally invasive thoracic surgery; a balanced mixture of an essay and a short state-of-the-art review was planned. It was intended to have a ‘*quo vadis*- where to go from here’ - red tag. Experience obtained from a series of endless postgraduate surgical training project planning meetings in different European countries was to be condensed. The main questions to be answered were as follows: 1) What are the established methods of video-assisted thoracic surgery (VATS) today? 2) How and when are these to be performed? This was thus to provide a perspective of our present practice with special emphasis on telesurgery - to make it short, I warned myself - a proper shopping list with an eye on the market and on the future.

I tried to resist the temptation of writing a hagiography on VATS. Let me cite the closing remarks of the final draft: ‘there is no such thing as a minimally invasive surgery. This *mantra* of ours is misleading. The level of surgical agressivity is not an independent factor. VATS has no right to be an aim of its own. What we should pursue, instead, is an optimally invasive operation, which of course should involve an adequate exposure commensurate to the patho-anatomical situation requirements. We should expect to pay a price for the proportional collateral damage in terms of pain, loss of function and scar - but at the lowest possible level. History of surgery teaches us that the patient and the disease are the Ka’aba stone around which everything else revolves in order to find their proper place. The biology of the pathological process and the rest of the body surrounding the diseased focus are the independent variables and by no means the surgical technicalities. It leads us nowhere if in focusing on the sharpeness of our knives the minimal arch of the blade is discussed. Having said that, no responsible surgeon takes the technical details lightly. Any self-respecting doctor considers that his/her paramount duty lies in obtaining the right balance between achieving maximum effectiveness without exposing the patient to undue harm or risk. Optimization of the agressiveness of surgery is the enigma of the art of surgery.[1] Walking the fine line between the Scylla of unwanted damage and the Charybdis of unnecessary compromises in surgical (oncological) correctness, we should define the proper place for VATS. Can we neglect the socioeconomic surroundings of the patient in this process? Definitely not.’ Expanding the horizons of the discourse beyond the limits of the surgical profession, I was ready to complete the final version; or so, I thought.

One week prior to the promised submission date, I was asked to join a rapid response catastrophe aid team (NGO) as a surgeon (nobody seemed to care too much about my credentials in chest surgery and, in particular, VATS methods) at a short notice. Having returned from the mission, I had no choice but to rethink and consequently rewrite the whole article.

In the refugee camps, even the very basic necessities were frequently lacking. A doctor’s duty was to provide relief to patients with possibly the simplest and commonest diseases as the cure was out of reach in many a cases. From that point of view, it seemed laughable and required a tunnelled vision to accept the importance and to discuss the number and length of port incisions. As it happened, not too much of my surgical skill was needed in Beirut/Lebanon, where we wandered around makeshift refugee camps and converted schools. Rural medicine, county surgery, basic pediatrics, scants of psychiatry and endless hours of public health medicine - that was our job in caring for these displaced people. I realized that in the same manner as the refugees benefited from my surgical experience within the whole spectrum of delivered medical care, the individual patient undergoing chest surgery too benefits from properly planned and executed procedures like VATS.

The quest for the role of VATS is emerging. Before proceeding beyond answering the questions that emerged - let me reflect briefly on the present situation.

Standards of VATS (laparoscopic surgery takes its roots in thoracic procedures)[2] are well established. Even the Veress needle comes from the pre-streptomycin tuberculosis era and was originally used for creation of pneumoperitoneum with therapeutical intent.[3] Exploration and biopsy of the mediastinum (collar, extended, Chamberlain mediastinoscopies), resection of solitary tumors and thymectomy, manipulations within the pleural space (pleural biopsy,[4] resection, pleurodesis and debridement) via VATS are all accepted and standardized procedures.

More and more procedures on the adjacent structures (spine, diaphragm: resection, suture; pericardium: exploration, windows)[5,6] involve video assistance. The limitations are mainly biological in that the patient should tolerate single-lung ventilation. The Nuss procedure and its modifications radically changed pectus surgery, leaving only the real challanges for those who are preferring the open access.

Diseases of the pulmonary parenchyma are totally different in this respect. In benign conditions, VATS parenchymal resections for diagnostic purposes and with therapeutic intent are considered justifiable, provided the patient can tolerate one-lung ventilation and a free intrapleural space can be found (frequently missing in post-inflammatory cases).

But there are two fields where serious concerns are emerging and these are beyond the mere technical feasibility. In fact, a well-trained chest surgeon is able to perform procedures identical to their open counterparts via thoracic ports using endotools - provided limitless theater time, budget and anesthesia are available. The question however is, Does it make any sense?

In malignant lung diseases, the obligatory hilar and mediastinal lymphadenectomy (lung cancer surgery) is hardly performable using VATS methods as safely, completely and quickly as via open access. Till unselected VATS lobar/sublobar resection with effective neoadjuvant therapy does not provide the same outcomes as standard lobectomy/ pneumonectomy using open thoracotomy, the limited access methods cannot expect full acceptance. More correct universally affordable and reliable imaging for proper preoperative staging is the other crucial obstacle[7] to overcome before the question of VATS as a standard in lung cancer surgery can be seriously considered.

Chest trauma is the other field where the controversies come to light. The problem is rooted in the taxonomy. Limiting the definition to the treatment of acute chest cases, the question that emerges is, Can we overcome the principal problems of trauma care - racing against time (the golden hour) and the inherent limitation of resources?

VATS is a time-consuming and multidisciplinary specialty - for example, a detailed preoperative checkup is performed by a pneumonologist (assessment of the functional reserve of the opposite lung) and the anesthetist has to be highly trained for single-lung ventilation via a double-lumen intubation. In chest injury, we are often faced with a patient the quality of whose lung parenchyma is unknown and who has a narrow threshold so far as the safe securing of airways is considered. The least desirable outcome is a surprise collapse of the ventilation on the table. It is hard to justify VATS in acute cases without violating the very norms of basic patient safety.

However, if we extend the definition of chest trauma surgery to include elective cases such as late pneumothorax and hemothorax, secondary empyema thoracis,[8] new vistas open up for the application of VATS techniques. But not respecting the borders or freely trespassing the limits just for the sake of using a given method on a new territory causes more harm than good.

This is where we are now. That is what we ought to teach. I cannot resist the temptation to compare ourselves (medical educators, teachers, doctors, professors) to generals of the military. Those generals are said to be excellent in preparing and training brilliantly their armies to fight and win the previous war. Training junior surgeons appears to be quite similar. How should we train our junior surgeons to perform procedures that are not yet fully discovered? How should we teach and practice unborn methods?

What we are able to transfer is the proper surgical attitude and the way of critical thinking. We can teach techniques, modern VATS methods included. But to show the residents when and when not to perform VATS is akin to guiding them in finding the proper path. Controlled mass experience - aka publication - is a pillar of wisdom full of bias. ’Publication fever’ masks the fact that frequently only a few enthusiastic centers (concentration of experts and resources) are genuinly able to present convincing and reproducible results. The main reasons are the extreme technology and resource dependencies of this field. Nobody seems to be keen to publish high failure rates or even the complication rates. Neither tumor surgery nor trauma care is a field where double-blind randomized trials can be carried out as they presently exist.

Quest for the best procedure in minimal access thoracic surgery should involve the answer to the question ‘*cui prodest*,- to whom do we serve?’ In this beautiful new world of consumer-satisfaction, guided medicine offering the best procedure is the *mantra*. It sounds quite obvious till one does not dare ask, What do we mean by ‘best’? Then we are back to square one - another problem with terminology.

Is surgery a service? The word derives from the Latin *servus*, meaning *servant. Ego sum servus tuus*- I am your servant. Sounds quite politically incorrect. But as surgeons, whom do we serve? Employees are serving their firm. The aim is to achieve the highest possible profit. The human beings they engage are called clients. Professionals are serving patients and their own profession. We should answer the recently presented question: Are we, as surgeons, employees or professionals?[9]

Where can we find surgeons - in the garden of consumer satisfaction - and who are the inhabitants of this supposed paradise? Are they the patients of the present? Are they the doctors? Or are they the invisible men sitting behind the till? The managers? Or are they those who pay their contribution to whatever funds or Health Insurance Company? One-day surgery is a typical case. There is a tremendous pressure to shorten the hospital stay.[10,11] The previously mentioned extra-medical factors are coming into the picture. Economy and politics are interfering with surgery in general and they have a heavy hand in minimal access surgery - a highly lucrative field for profit. Hospitals and doctors are expected to follow and copy the patterns of the industry - our art is frequently getting referred to as ‘health industry.’

We are frequently reminded of a constant race for excellence. But one must ask, Is it really for excellence? What do we mean by excellence and how do we measure it? How wide is the gap between patient satisfaction and real value of care by means of outcomes and who is bearing the burden of cost? Our anesthetist colleagues are using more and more advanced high technology anesthesia machines - with functions rarely fully understood and used. I was told that less than one-third of the capacity is utilized. There is no need to go so far - to make excursions on the other side of the isolation screen. On our side of the screen, what proportion of functions of an expensive and sophisticated electrodiathermy device do we actually use?

Perpetually demanding further resources is a sin shared by all the participants. The ‘tool spiral’ is a more than worrying example. Stapler prices, single-use tools are draining the budgets. Who remembers the good old Russian staplers - thoracic equivalents of the Petz gastric resection machine[12] with their reloadable magazines/cartridges? Are we really better with all our brand-new gadgets? Are all of them really necessary and if so at what cost? Is there a (golden) middle path avoiding extremes? Did we resist the temptations or just accepted the push for more advanced, more sophisticated machines, created by a market with a huge suction force, without caring for the consequences? Did we do our share of job by adjusting our needs to the economical capacities of our countries and societies?

Where do we go from here? No one can ignore the influence of the surrounding world. Nobody expects us to stand up and defend the trenches till the last endostapler is fired. We must listen to the message of our past.[13] Never to bow to extra-professional pressures should remain one of our chief commandments. If morale, philosophy and theory construct always, if subliminally, the basics of practice of surgery, we should be able to remain responsible professionals in a globalized world, which at the time tends to de-professionalize more and more territories. It is only by strongly committing to our patients as individuals and as surgeons concerned with optimization of the level of aggressiveness can we protect them and ourselves.
